# The relation of dietary components with severity of obstructive sleep apnea in Cypriot patients: A randomized, stratified epidemiological study

**DOI:** 10.1371/journal.pone.0265148

**Published:** 2022-03-11

**Authors:** Stavri Chrysostomou, Frangiskos Frangopoulos, Yiannis Koutras, Kosmia Andreou, Lydia Socratous, Konstantinos Giannakou

**Affiliations:** 1 Department of Life Sciences, School of Sciences, European University Cyprus, Nicosia, Cyprus; 2 Respiratory Department, Nicosia General Hospital, Nicosia, Cyprus; 3 Department of Health Sciences, School of Sciences, European University Cyprus, Nicosia, Cyprus; Sapienza University of Rome, ITALY

## Abstract

**Background:**

Obstructive Sleep Apnea (OSA) is considered a public health problem and its prevalence is increasing at an epidemic rate. The aim of this study was to examine whether individual nutrients (macronutrients, antioxidant vitamins) rather than energy restriction may potentially affect OSA severity in a representative population of Cyprus.

**Methods:**

A total sample of 303 adults (>18 years old) with Cypriot citizenship and permanently residing in Cyprus were randomly selected. Selected patients have completed the food frequency questionnaire, and a physical activity questionnaire and underwent a sleep study to assess OSA severity.

**Results:**

Overall, 303 patients were included in this study, 169 (55.8%) had mild OSA (apnea-hypopnea index—AHI <15) and the remaining 83 (27.4%) had moderate to severe OSA (AHI>15). The mean age of all patients was 55.7 years old. Patients with moderate to severe OSA had significant higher BMI levels, higher consumption of calories, higher hip circumference, waist circumference, waist-hip ratio and neck circumference and higher consumption of folic acid compared with the patients with mild OSA (p<0.05).

**Conclusions:**

The findings suggest that increased energy intake regardless diet macronutrient composition is positively associated with OSA severity whereas higher folic acid intake seems to have a protective role.

## Introduction

Obstructive sleep apnea (OSA) is characterized by upper airway obstruction during sleep, resulting in recurrent oxidative stress and sleep fragmentation [1]. The severity of OSA is determined by the Apnea-hypopnea index (AHI), where a value of 5 to 14.9 breathing disturbances per hour of sleep is considered a mild burden, 15 to 29.9 is considered as moderate, and an AHI of 30 or greater is considered severe [2]. The prevalence of OSA is increasing at an epidemic rate. Approximately 25% of men and 13% of women suffer from OSA worldwide [3]. A recent population-based study in Cyprus estimated that the prevalence of moderate to high risk for OSA is 50% in males and 18% in females [4].

OSA is considered a key public health problem as it associated with an increased risk of non-communicable diseases [2, 5]. Epidemiological studies reported that severity of OSA is associated with insulin resistance and type 2 diabetes mellitus, dyslipidemia, hypertension, and increased cardiovascular and all-cause mortality [6–8]. A previous study demonstrated that predictors of cardiovascular diseases such as the gamma glutamyl transferase (GGT), significantly increased in a proportional manner as the severity of OSA increased indicating a strong association between cardiovascular events in patients with OSA [9]. Thus, increased public awareness and early diagnosis of OSA is critical to reducing cardiovascular disease burden and other complications.

Based on the literature, there is a strong association between inflammation, sleeping disturbances and OSA. Intermittent hypoxemia and sleep fragmentation result in increased proinflammatory status with oxygen free radicals, cell free DNA and several increased cytokines [10, 11]. In particular, chronic inflammation associated with the pathology of OSA involves the production of proinflammatory cytokines, oxygen free radicals, lipid peroxidation products and consequent formation of atheromatous plaque [12]. Both in children and adolescents, OSA is accompanied by a chronic inflammatory state which is also present in the nasal mucosa. It has been found that rhinosinusitis is allergic but also vasomotor associated with obstructive sleep disorders although the nose does not represent a site of collapse of the upper airways. However, the treatment of rhinitis certainly reduces snoring and increases CPAP compliance [13, 14]. Moreover, evidence in the literature also describes a correlation between olfactory disturbances and OSA with a linear correlation between OSA severity and loss of smell whereas the CPAP treatment could reverse the olfactory function [15, 16].

OSA is a complex multifactorial disease influenced by unmodifiable and modifiable factors [2]. Among the later, diet appears to be an important contributor [17]. In fact, lifestyle interventions are being encouraged for the treatment of OSA as a low-cost and easy-to-use treatment modality [18]. Many studies suggest that obesity is probably the strongest predictor of OSA [1, 2, 19]. Obesity is characterized by an increased adipose deposition within the human body and commonly around pharynx (neck) causing increased tissue pressure (mechanical load) and arousals during sleep [20]. Moreover, it is recognized that pro-inflammatory cytokines are secreted from adipose tissue [21]. In this case, cytokines may depress neuromuscular control of the upper airway and induce the production of active oxygen species [22]. These oxygen species decrease the force-generating capacity of skeletal muscle and negatively affect the upper airway function leading to upper airway neuropathy [23].

A healthy diet includes an abundant molecule with antioxidant and anti-inflammatory properties, such as omega-3 unsaturated fatty acids, folic acid, vitamins A, C and E, phenolic compounds, and fibers, which, when consumed in combination may have synergistic effects [24]. The anti-inflammatory and antioxidant effect of these molecules may benefit patients with OSA possibly by improving upper airway neuromuscular control and upper airway muscle force-generating capacity [1]. The influence of antioxidant nutrients such as Vitamins A, C, E, folic acid on cognitive decline and cardiovascular diseases have been previously examined [25]. However, the influence of antioxidant vitamins on OSA remains to be investigated. Moreover, among the dietary interventions for the treatment of OSA, other theories support that a low carbohydrate intake results in a greater reduction in pro-inflammatory cytokines and thus improving the upper airway neuromuscular control in obese patients with OSA [26]. However, most of these studies employed calorie-restricted diets to induce weight loss [27] and thus, the therapeutic potential of individual nutrients on OSA is not clear [28].

Cumulative evidence has supported that diet could be a promising approach for improving OSA severity. However, it is yet not clear whether potential improvement is due to energy restriction and induced weight loss or due to diet composition in terms of macronutrients and micronutrients. To date, most of the studies examined the effect of several calorie-energy restricted diets on OSA severity ignoring the possible effects of individual dietary components. Nevertheless, identifying effective nutrients that may have a potential on OSA severity is an important issue for public health practice considering the high social and economic impact of the disease. Therefore, the aim of this study was to examine whether individual dietary components (macronutrients and antioxidant vitamins) rather than calorie-energy restriction may potentially affect OSA severity in a representative sample of population of patients in Cyprus.

## Methods

### Study population

The study was conducted in conjunction to the OSA epidemiological study in Cyprus [4]. The reporting of the study follows the Strengthening the Reporting of Observational Studies in Epidemiology statement [29]. Eligible participants for inclusion in the study were adults age > 18 years old with Cypriot citizenship and permanently residing in Cyprus and willing to participate to the study. To ensure the representativeness of the sample, the sample was stratified according to the last demographic report (2016) by district, rural or urban area, gender, and age. Stratification ensures that the sample is representative of the population with respect to the chosen population parameters. This study was conducted according to the Declaration of Helsinki guidelines, and all procedures involving research study participants were approved by the Cyprus National Bioethics Committee (CNBC) (EEBK/EP/2016/35). All participants signed a written consent to participate in the study after given all the appropriate information.

All willing participants (N = 4118) were interviewed by phone and answered the STOP-Bang questionnaire as previously described [30]. The STOP-Bang questionnaire is an easy to-use, self-reportable, screening tool for OSA that includes eight questions of yes/no about snoring, tiredness, observed apnea, high blood pressure, BMI>35 kg/m^2^, age>50 years old, neck circumference > 40 cm, and male gender [30].

**Fig 1:**
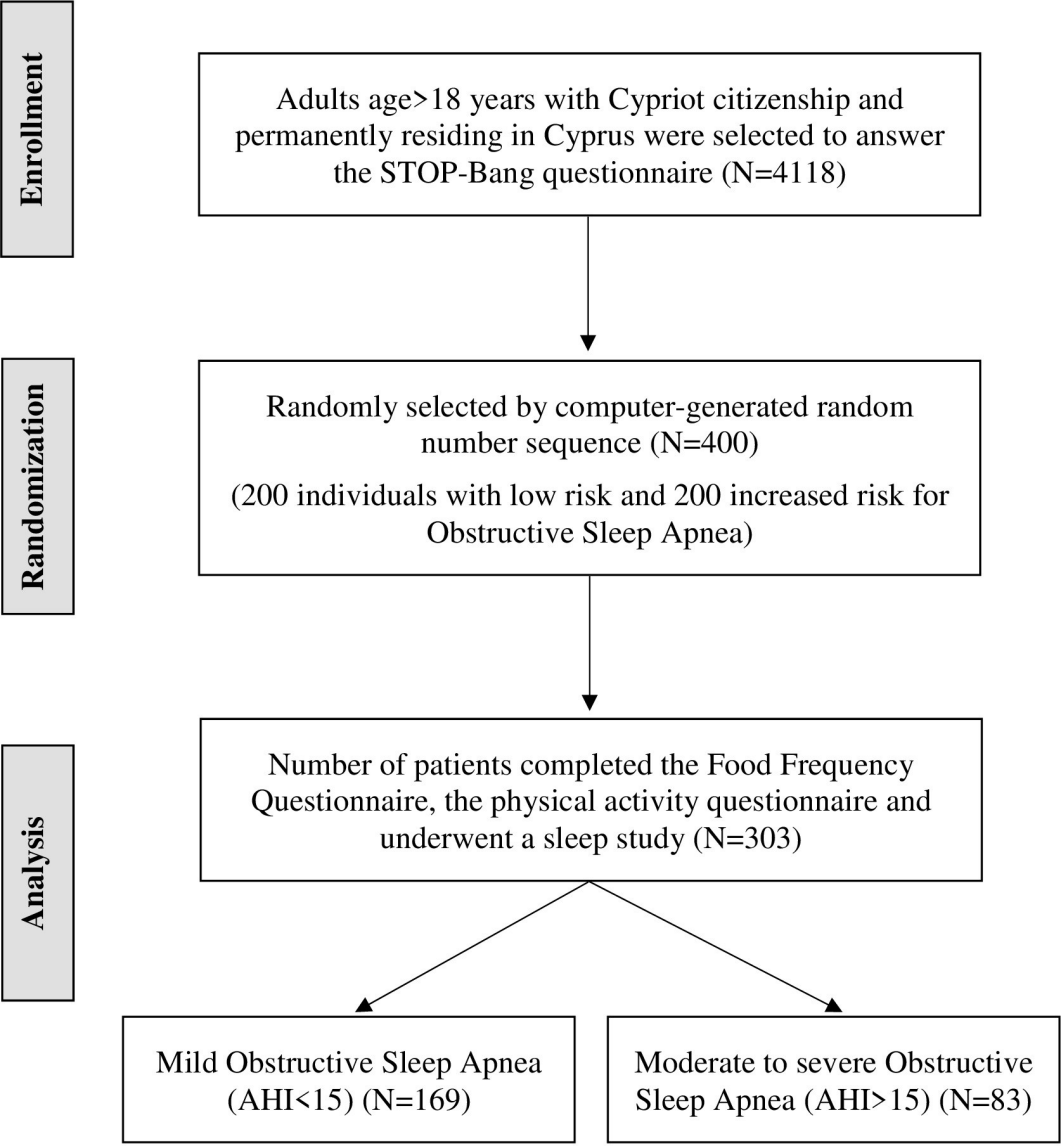
Study selection flow diagram.

A secondary cross-sectional nationwide survey was piloted to examine the possible associations between the characteristics of sleep-disordered breathing measured by a type III sleep test and diet. A type III sleep testing device monitors a minimum of four channels that include one or more channels of respiratory effort, airflow, oxygen saturation, and heart rate/electrocardiogram. Consequently, from the initial representative sample of the Cypriot adult population (4118 participants), 400 adults (200 low risk and 200 increased risk for OSA, based on the STOP-Band questionnaire) were randomly selected to participate in the second stage procedure (computer-generated random number sequence). Among these participants, 303 completed the Food Frequency Questionnaire (FFQ), the physical activity questionnaire and underwent a sleep study (response rate = 76%). The study selection flow diagram is shown in **Fig 1**.

### Anthropometric measurements

An easily calibrated precision scale was used for weight measurement. Weight was measured to the nearest 10 g with participants wearing light clothing and no shoes and they also attempted to empty their bladder. Height was measured in the standing position using a stadiometer accurate to ±0.5cm. Body Mass Index (BMI) was calculated using the standard equation (kg/m2). (kg/m^2^). Neck circumference (NC) was measured at the middle of the neck between the mid-cervical spine and superior line of the cricothyroid membrane in a standing position. Waist Circumference (WC) was measured between the lowest rib and the crista iliac superior and Hip Circumference (HC) was measured as the maximum circumference of the buttocks. Waist-hip ratio (WHR) was obtained by diving WC (cm) by HC (cm) [31].

### Food Frequency Questionnaire (FFQ)

A quantitative FFQ was administrated to all participants undertaken the type III sleep test. The FFQ was based on typical foods and contained 68 items and 13 sub-questions. Participants were asked about the frequency of intake of several types of food during the last month. Also, they were asked to report the frequency of these intakes in terms of day, week or month [32]. All data were analyzed in terms of total daily total energy, macronutrient (proteins, carbohydrates, sugars, total fat, saturated fat, polyunsaturated fat, monounsaturated fat and fibers) and antioxidant vitamins intake (vitamins A, C, D, E, and folic acid) by using the dietary analysis software SNPRO Nutrition Software (Cheapsoft Softwares, 2017).

### Assessment of Physical Activity (PA)

A questionnaire about PA was administered to all eligible participants together with the FFQ. Participants were questioned about the frequency, intensity and duration of their PA and based on these three parameters the PA index was calculated. Estimated levels of PA were defined based on the total scoring of the index as Sedentary Activity (<14), Low to Moderate Activity (15–40) and Activity to High activity (>41) [33, 34].

### Statistical analysis

Baseline characteristics of the participants are reported as mean ± standard deviation (SD) for continuous variables with normal distributions and as median interquartile range (IQR) for continuous measures with skewed distributions while categorical variables were presented as absolute (n) and relative (%) frequencies. Shapiro-Wilk test was used to check if numeric variables were normally distributed. OSA severity categories were defined by AHI cut-offs: AHI <15 was categorized as ’’Mild OSA’’, and AHI>15 as ’’Moderate and severe OSA’’. To detect any differences among OSA categories and the categorical demographic and physical characteristics of the participants, Pearson’s chi square test was used. T-test for normally distributed variables and the Kruskal-Wallis rank test for non-normally distributed variables were used to assess any differences between OSA categories and continuous characteristics of the participants. All statistical tests performed were two-sided with statistical significance level set at α = 0.05. Statistical analysis was conducted using SPSS Statistics v. 26.0 (IBM, Somers, NY, USA).

## Results

### Participants’ demographics and physical characteristics

**Table 1** summarizes demographic and physical characteristics of the total sample and divides the patients according to the categories of AHI representing severity of OSA. A total of 303 patients participated in the study. Among the total sample, 169 (55.8%) patients had mild OSA (AHI<15) and the remaining 83 (27.4%) had moderate to severe OSA (AHI>15). The mean age of all patients was 55.7 (SD = 12.9) years old. Among all the patients, almost 69% were males, most of them had sedentary lifestyle (73%), and approximately 76% were categorized as overweight or obese. The median hip perimeter was 109 (IQR = 14), whereas the mean waist circumference and neck circumference were 102.7 (SD = 15.2) and 41 (SD = 4.6), respectively. The mean WHR was 0.93 (SD = 0.09) (**Table 1**).

**Table 1:** Demographic and physical characteristics of the patients according to the categories of apnea hypopnea index representing severity of obstructive sleep apnea (n = 303).

Characteristic	Total	Mild OSA (AHI<15) (n = 169)	Moderate to severe OSA (AHI>15) (n = 83)	P value
**Gender**, n (%) (n = 253)				**0.028**
*Male*	174 (68.8%)	109 (63.0%)	64 (37.0%)	
*Female*	79 (31.2%)	60 (75.9%)	19 (24.1%)	
**Age**, years (mean ± SD) (n = 253)	55.7 ± 12.9	53.6 ± 13.1	60.0 ± 11.4	**<0.001**
**Physical activity levels**, n (%) (n = 125)				0.096
*Sedentary (<14)*	91 (72.8%)	42 (56.8%)	32 (43.2%)	
*Low to moderate activity (15–40)*	21 (16.8%)	13 (76.5%)	4 (23.5%)	
*Activity and high activity (>41)*	13 (10.4%)	10 (83.3%)	2 (16.7%)	
**BMI (kg/m**^**2**^**)**^**1**^ (n = 253)	28.37 ± 7.3	26.81 ± 6.0	31.56 ± 7.1	**<0.001**
**BMI categories**, n (%) (n = 252)				**<0.001**
*Underweight (<18*.*5 kg/m*^*2*^*)*	2 (0.8%)	2 (100%)	0 (0%)	
*Normal (18*.*5 to 24*.*9 kg/m*^*2*^*)*	59 (23.4%)	49 (83.1%)	10 (16.9%)	
*Overweight (25 to 29*.*9 kg/m*^*2*^*)*	97 (38.5%)	73 (76.0%)	23 (24.0%)	
*Obesity (>29*.*9 kg/m*^*2*^*)*	94 (37.3%)	44 (46.8%)	50 (53.2%)	
**Energy (calories)**^**1**^(n = 302)	2434 ± 1284	2465 ± 1329	2703 ± 1469	**0.046**
**Hip circumference (cm)**^**1**^ (n = 199)	109.0 ± 14	107 ± 12	112 ± 15	**<0.001**
**Waist circumference (cm)** (mean ± SD) (n = 199)	102.7 ± 15.2	99 ± 13.8	110.7 ± 15.3	**<0.001**
**Waist-hip ratio** (mean ± SD) (n = 199)	0.93 ± 0.09	0.91 ± 0.09	0.96 ± 0.08	**<0.001**
**Neck circumference (cm)** (mean ± SD) (n = 251)	41.03 ± 4.6	39.9 ± 4.1	43.3 ± 4.6	**<0.001**

Abbreviations: AHI, apnea-hypopnea index; BMI, body mass index; IQR, interquartile range; OSA, Obstructive Sleep Apnea; SD, Standard Deviation.

^1^Data are expressed as median ± IQR.

### Demographic and physical characteristics according to the categories of OSA

The mean age of the 169 patients who had mild OSA was 53.6 years (SD = 13.1), whilst the mean age of the 83 patients with moderate to severe OSA was 60 years (SD = 11.4) (p<0.001). We observed statistically significant differences between the two groups as regards to the BMI. Specifically, we noticed a larger median BMI among those patients who had moderate to severe OSA compared to the patients with mild OSA (p<0.001). Patients with moderate to severe OSA had significant higher consumption of calories compared to the other group (p<0.05). In addition, hip circumference, waist circumference, WHR and neck circumference were significantly higher in OSA patients with moderate to severe OSA compared with the patients with mild OSA (p<0.001 for all). No statistically significant difference was observed between both groups of patients regarding the physical activity levels (p = 0.096).

### Nutritional intake according to the categories of OSA

**Table 2** presents the nutritional intake of the total sample and across the categories of severity of OSA. Patients with mild OSA had statistically significant higher consumption of folic acid compared to the patients with moderate to severe OSA (p<0.05). No statistically significant differences were observed between both groups of patients among all the remaining individual nutritional characteristics (p>0.05).

**Table 2:** Nutritional intake across categories of apnea hypopnea index representing obstructive sleep apnea (n = 303).

Characteristic (mean ± SD)	Total	Mild OSA (AHI<15) (n = 169)	Moderate and severe OSA (AHI>15) (n = 83)	P value
**Carbohydrate (gr)**	340.49 ± 186.1	330.14 ± 181.2	376.02 ± 218.7	0.102
**Sugars (gr)**	5.16 ± 7.49	4.58 ± 6.19	6.43 ± 10.5	0.228
**Fibre (g)**	45.65 ± 34.8	43.23 ± 31.4	52.79 ± 46.3	0.092
**Proteins (gr)**	96.80 ± 59.2	96.30 ± 50.7	104.60 ± 77.4	0.309
**Fat (gr)** ^**1**^	89.65 ± 65.5	93.0 ± 66.5	88.0 ± 70.5	0.930
**Saturated Fat (gr)** ^**1**^	25 ± 21.8	24 ± 20.8	27 ± 22.5	0.601
**Polyunsaturated Fat (gr)**	16.42 ± 12.5	16.33 ± 11.8	16.01 ± 13.4	0.847
**Monounsaturated Fat (gr)** ^**1**^	42.68 ± 32.1	45.0 ± 30.7	42.7 ± 38.8	0.906
**Vitamin D (μg)** ^**1**^	14.39 ± 19.6	13.99 ± 18.0	13.09 ± 36.8	0.500
**Vitamin C (mg)**	380.28 ± 339.4	380.28 ± 339.4	380.28 ± 339.4	0.153
**Vitamin A (μg)** ^**1**^	360.15 ± 529.2	360.15 ± 560.9	360.15 ± 576.2	0.236
**Vitamin E (mg)** ^**1**^	0.28 ± 0.86	0.29 ± 0.91	0.27 ± 0.85	0.729
**Folic acid (μg)** ^**1**^	0.91 ± 7.9	1.14 ± 10.8	0.09 ± 8.0	**0.047**

Abbreviations: AHI, apnea-hypopnea index; BMI, body mass index; IQR, interquartile range; OSA, Obstructive Sleep Apnea; SD, Standard Deviation.

^1^Data are expressed as median ± IQR.

## Discussion

The current community-based study of 303 subjects was conducted in Cyprus and subjects were divided in two groups according to AHI levels. Patients with moderate to severe OSA were significantly older, with higher levels of BMI, higher energy intake and were characterised with central obesity compared to patients with mild OSA. Regarding nutrient intake, it seems that macronutrient intake was similar among the two groups, but folic acid intake was significantly higher in patients with moderate to severe OSA compared to patients with mild OSA.

NC, WC and WHR are clinical features of central obesity shown by several studies to be associated with OSA [35, 36]. Previous studies indicated a relationship between OSA and anthropometric markers, but their results have been challenged. In addition, previous findings revealed that increasing WC over adult life has a stronger association with OSA severity compared to NC [37, 38]. A different study indicated that NC and BMI measurements were higher in severe OSA patients compared to non-severe patients and that increased NC may be a greater risk factor for severe OSA than WC [35]. In contrast, the study by Martinez-Rivera et al. (2008) found that WHR but not BMI was associated with OSA severity [39] while the study by Davidson and Patel (2008) concluded that WC is a better measure than BMI or NC to predict OSA [40]. On the other hand, our study suggested that all included markers of central obesity (NC, WC and WHR) were significantly higher in patients with moderate to severe OSA compared to patients with mild OSA. A reasonable explanation for conflicting results reported in the literature might be due the different AHI cut-of points used for the determination of OSA severity in each study. In fact, there are several mechanisms explaining the great importance of fat accumulated in the abdomen and neck area compared to the peripheral in patients with OSA [36]. Thereby, our findings strongly support those patients with central obesity determined by increased levels of NC, WC and WHR are identified as high-risk patients for severe OSA and therefore, future preventing strategies for OSA should focus among them.

The benefits of weight-loss interventions programs on OSA are established in previous clinical trials [27, 41]. Our study also agrees with previous findings showing that the amount of calories consumed is positively associated with the severity of OSA. However, most of the previous trials mainly focused on the caloric content of each diet aiming for weight loss without considering the potential effect of individual dietary components on OSA. In our study, none of the macronutrient’s intake was associated with OSA severity and this seems to conflict with previous findings. Particularly, recent studies reported that diet composition and diet quality is an important mechanism linking obesity to Sleep Disturbances (SD) [42] probably through the effects of specific nutrients on inflammation, hormonal responses involved in hunger-satiety mechanism and energy metabolism [43]. Moreover, Hargens et al. reported that dietary fatty acids including n-3 and n-6 poly-unsaturated fatty acids are involved in sleep regulation [42], through their effect on some biochemical compounds which exert an important role for the initiation and maintenance of sleep [44, 45]. In addition to fat, high carbohydrate consumption and a low quality diet were associated with increased SD [42, 46] whereas fiber consumption was associated with better quality of sleep [47]. Moreover, a diet low in fatty acids and carbohydrates allows a reduction in body weight, circulating lipids and chronic inflammatory state related to obesity. Therefore, the modified lifestyle represents a useful therapeutic option, to be associated in all cases with medical treatment [48, 49]. However, the above studies aimed to explore associations among dietary food components and SD in other population and not particularly for OSA. Although OSA is characterised by SD, it is through a separate clinical condition and thus future research should aim to examine the effect of these dietary molecules particularly in patients with OSA.

The current literature is limited regarding the role of antioxidant vitamins in OSA pathogenesis. Consequently, it is uncertain whether increased antioxidant nutrient intake or supplementation would improve OSA status per se [25]. In regards to antioxidants intake, our study indicated that intake of antioxidant vitamins A, C, D and E was not associated with OSA severity. Although previous studies showed a relationship between OSA and antioxidant response, their results have been conflicting. Some authors provided findings suggesting that OSA patients had lower antioxidant levels [50, 51]. The study by Singh et al. found that antioxidant glutathione levels decreased in OSA patients but got restored after oral antioxidant supplementation (vitamin E 400 IU and vitamin C 100 IU for 45 days) [52]. In addition, a previous study revealed that that the endothelial dysfunction in OSA patients was reversed after intravenous injection with vitamin C [53]. In contrast, some other studies showed no significant differences [54, 55]. In this respect, the relationship between OSA and antioxidant vitamins remains unclear and requires further research.

There is accumulating evidence for an increased oxidative stress in OSA. Previous studies reported enhanced in vitro release of free oxygen radicals in patients with OSA [56] and reduced plasma levels of nitric oxide (NO) derivatives [57, 58]. Notably, our study indicated that folic acid intake was significantly higher in patients with moderate to severe OSA compared to patients with mild OSA. Folic acid is a water-soluble B-complex vitamin, associated with improved endothelial dysfunction. In particular, the folate metabolite, 5-methyltetrahydrofolate, has the ability to promote NO production and also serve as a superoxide radical scavenger [59]. Therefore, the most likely explanation for our findings is that folic acid could improve OSA severity through its antioxidant activity and that lower intake of folic acid increases oxidative stress and inflammation leading to severe OSA. Recent studies demonstrated that folic acid plays an important role in preventing diseases related to oxidative stress [60]. A previous study indicated that folic acid supplementation improved sleep deficiency-related disorders improving oxidative stress [61]. In line with our findings, the study by Baldwin et al. reported reduced dietary intake of folic acid, and vitamins C and E for subjects diagnosed with OSA compared to subjects without OSA [62]. However, relative studies examining the effect of folic intake or supplementation on OSA are scarce and further studies are required in order to conduct more concrete conclusions.

Treating obesity through energy restricted diets is one of the most effective strategies for OSA prevention and management. However, data regarding the effect of diet composition on OSA treatment is limited. Our study indicated that increased energy intake regardless diet macronutrient composition is positively associated with OSA severity. Regarding antioxidants vitamins, it seems that only folic acid intake is inversely associated with OSA severity exerting a possible protective role. However, research on antioxidant vitamins in OSA is still in very early stages and therefore, future research should focus on nutrient and supplement intake to determine whether higher intake of folic acid or even other nutrient or antioxidant contributes to better OSA outcomes, and whether nutritional supplementation can decrease OSA-associated complications. Further studies are also needed to fully elucidate the association and mechanisms of individual macronutrients and antioxidant vitamins in OSA and any need for supplementation. Indeed, a previous study indicated that aadequate sleepers had better serum-measured inflammation, oxidative stress, and antioxidants profiles and that some antioxidant vitamins such as carotenoids, vitamins C and D are modest mediators of the sleep duration. It seems that the promotion of adequate sleep duration could improve inflammation, oxidative stress and antioxidant capacities and prevent or improve cardiometabolic dysfunction [63]. However, further longitudinal studies are needed to clarify the molecular pathways that exist beyond this correlation.

### Strengths and limitations of the study

To the best of our knowledge, this is the first nationwide study that examined the relation of diet with OSA severity in Cypriot patients. Moreover, the randomized selection of the community-based sample identified an optimal sleep lab-naïve sample. Also, there is no referral bias since all subjects were recruited from the community rather than the clinic. Potential limitations should be acknowledged. In particular, the observational nature of this study indicates associations, and no causation can be drawn. Another limitation of this study is that all data derived from questionnaires were self-reported, a fact which could potentially lead to misreporting and information bias. Unmeasured factors, such as smoking habits, were not assessed and confounding by these variables cannot be excluded. Moreover, sex differences were not assessed in the study due to the confounding sample size, which included only 31.2% females probably due to the low prevalence of OSA among females. Lastly, the study includes mainly Caucasians, and thus the findings might not be generalizable to other populations.

## Supporting information

S1 Dataset (SAV)
